# Technical Validation of a Hepatitis C Virus Whole Genome Sequencing Assay for Detection of Genotype and Antiviral Resistance in the Clinical Pathway

**DOI:** 10.3389/fmicb.2020.576572

**Published:** 2020-10-09

**Authors:** Carmen F. Manso, David F. Bibby, Kieren Lythgow, Hodan Mohamed, Richard Myers, David Williams, Renata Piorkowska, Yuen T. Chan, Rory Bowden, M. Azim Ansari, Camilla L. C. Ip, Eleanor Barnes, Daniel Bradshaw, Jean L. Mbisa

**Affiliations:** ^1^Public Health England, London, United Kingdom; ^2^The Wellcome Centre for Human Genetics, University of Oxford, Oxford, United Kingdom; ^3^Peter Medawar Building for Pathogen Research and the NIHR Oxford Biomedical Research Centre, University of Oxford, Oxford, United Kingdom; ^4^National Institute for Health Research Health Protection Research Unit (NIHR HPRU) in Blood Borne and Sexually Transmitted Infections, London, United Kingdom

**Keywords:** hepatitis C virus, antiviral resistance, genotyping, whole genome sequencing, next generation sequencing, direct acting antivirals, target enrichment, technical validation

## Abstract

Choice of direct acting antiviral (DAA) therapy for Hepatitis C Virus (HCV) in the United Kingdom and similar settings usually requires knowledge of the genotype and, in some cases, antiviral resistance (AVR) profile of the infecting virus. To determine these, most laboratories currently use Sanger technology, but next-generation sequencing (NGS) offers potential advantages in throughput and accuracy. However, NGS poses unique technical challenges, which require idiosyncratic development and technical validation approaches. This applies particularly to virology, where sequence diversity is high and the amount of starting genetic material is low, making it difficult to distinguish real data from artifacts. We describe the development and technical validation of a sequence capture-based HCV whole genome sequencing (WGS) assay to determine viral genotype and AVR profile. We use clinical samples of known subtypes and viral loads, and simulated FASTQ datasets to validate the analytical performances of both the wet laboratory and bioinformatic pipeline procedures. We show high concordance of the WGS assay compared to current “gold standard” Sanger assays. Specificity was 92.3 and 96.1% for AVR and genotyping, respectively. Discordances were due to the inability of Sanger assays to assign the correct subtype or accurately call mixed drug-resistant variants. We show high repeatability and reproducibility with >99.8% sequence similarity between sequence runs as well as high precision for variant frequency detection at >98.8% in the 95th percentile. Post-sequencing bioinformatics quality control workflows allow the accurate distinction between mixed infections, cross-contaminants and recombinant viruses at a threshold of >5% for the minority population. The sequence capture-based HCV WGS assay is more accurate than legacy AVR and genotyping assays. The assay has now been implemented in the clinical pathway of England’s National Health Service HCV treatment programs, representing the first validated HCV WGS pipeline in clinical service. The data generated will additionally provide granular national-level genomic information for public health policy making and support the WHO HCV elimination strategy.

## Introduction

Nucleic acid sequencing assays have been used for direct patient care in clinical virology for decades, for example, to inform management of antiretroviral therapy in HIV-infected patients (Hanna and D’aquila, 2001; Haubrich and Demeter, 2001). Conventionally, this involves targeted amplification of specific viral genes followed by sequencing using Sanger or recently, next-generation sequencing (NGS) technologies. However, this approach has limitations when used for highly diverse viruses such as hepatitis C virus (HCV), including the challenge of developing pan-genotypic target specific amplification assays. Thus, most available in-house and commercial assays are genotype-specific and do not cover all known and unclassified subtypes or recombinant virus strains (Rodriguez et al., 2018, 2019). For antiviral resistance (AVR), the assays require prior knowledge of the subtype of infecting virus and, as antiviral drugs target several viral gene products, multiple assays must be performed, resulting in a stepwise process that is laborious and not amenable to high throughput.

Recently, we showed that genotype-agnostic HCV whole genome sequencing (WGS) assays using sequence capture target enrichment provide genotype and AVR information in a single test and are amenable to high throughput (Thomson et al., 2016). Genetically, HCV is highly variable and, based upon nucleotide sequence, is divided into eight genotypes (1–8) which in turn are further subdivided into >90 subtypes (designated by letters of the alphabet e.g., 1a for genotype 1 subtype a) (Smith et al., 2014, 2019; Borgia et al., 2018). HCV strains belonging to different genotypes differ at 30–35% of nucleotide sites whereas strains that belong to the same subtype differ at <15% of nucleotide sites. The target enrichment probe baits used for the sequence capture assay are ∼120-bp long and can tolerate up to 20% mismatch in target sequence allowing intra-subtype, and in conserved regions, inter-genotype coverage; therefore, a small number of pooled probe baits can be used to cover the high diversity observed in HCV and detect unclassified subtypes (Bonsall et al., 2015).

Clinically, it is well established that there are genotype- or subtype-specific differences in response to treatment and knowledge of the genetic composition of a patient’s HCV strain is consequently of key importance for selecting the optimal treatment regimen (European Association for the Study of the Liver, 2018). Historically, HCV drug therapy was limited to pegylated interferon-α and ribavirin, which were more effective against genotypes 2 and 3 than other genotypes, required a prolonged course sometimes exceeding a year, and were associated with considerable toxicity. Furthermore, outcomes were often poor, due to a sustained virological response (SVR) of less than 50% in genotype 1-infected patients (Manns et al., 2001). New direct acting antivirals (DAA) directly inhibit the function of viral NS5a, polymerase (NS5b) or protease (NS3) and have minimal side effects, shortened duration of therapy of weeks, and yield SVR rates of >95% (Kohli et al., 2014). Notwithstanding the high efficacy of DAA, many do not have a pan-genotypic action and have mainly been evaluated against the common subtypes prevalent in the Western world, these being 1a, 1b, and 3a. Furthermore, the current NHS England HCV treatment rate card is predominantly genotype- and disease stage-specific, and therefore HCV genotyping is a prerequisite for optimal regimen determination (Hawkes, 2019).

The determination of HCV genotype has mostly been performed using line probe-based assays, real-time PCR assays, or partial genome sequencing of 5′UTR, core and/or NS5b domains. The third approach has been shown to be more accurate, but occasionally misassigns the subtype, and cannot identify recombinants unless several targets are used (Weck, 2005; Cai et al., 2013). These approaches are also unable to identify mixed infections. Additionally, drug resistance testing is recommended for the detection of key polymorphisms in genotype 1a viruses that have a strong effect upon response to the NS5a inhibitor elbasvir at baseline (Bradshaw et al., 2019). AVR testing is frequently requested by clinicians to guide retreatment strategy following treatment failure (Panel, 2018; Bradshaw et al., 2019). Thus, the determination of genotype and/or AVR at patient entry into care has clear and direct benefits. It enables early selection of the most appropriate regimen with concomitant reduction in the cost of treatment by avoiding the use of inferior or more expensive options, or retreatment. Furthermore, WGS provides refined genotyping data compared to current partial genome sequencing and can detect where a patient is infected with multiple strains or with a recombinant strain, data that are not generated by current techniques.

There are unique quality assurance challenges posed by viral NGS assays that often go unheeded and a paucity of standardized technical validation protocols essential to fulfill laboratory accreditation and regulatory requirements prior to implementation in the clinical pathway. Here, we perform the technical validation of a sequence capture HCV WGS assay implemented for direct patient care in the United Kingdom. We establish the quality metrics for consistent generation of robust HCV sequence data as well as determine the analytical performance of the assay. We also establish quality control procedures for detection and distinction between cross-contaminants, mixed infections and recombinant viruses which are significant quality assurance challenges faced by viral NGS assays.

## Materials and Methods

### Samples and Controls

Plasma samples submitted to Public Health England (PHE) for HCV genotypic and/or drug resistance analysis were selected for technical validation of the HCV Sequence Capture assay described in this study. Samples comprising a variety of genotypes and subtypes circulating in the United Kingdom and worldwide, and a range of viral loads were included in the validation of the assay.

Each MiSeq run was loaded with uniquely indexed, pooled libraries constructed from 72 samples: 62 clinical samples, two positive controls (PC), four negative human plasma (NHP, provided by NHS Blood and Transplant) controls, and four “no template controls” (NTC) of molecular grade water, were extracted. Both sets of negative controls were expected to be negative by HCV qPCR. The NHP controls were expected to have a total library mass yield similar to other clinical samples included in the experiment. In contrast, the total mass of the NTC libraries was expected to be no larger than background measurements.

To monitor cross-contamination within or between experiments, the positions of the control samples was shifted between consecutive batches of 72 libraries, and two independent sets of indexes were used in alternate runs.

### Library Preparation

For each sample, 350 μl of plasma was extracted using automated NucliSens easyMAG (BioMérieux) kits and eluted into a volume of 25 μl elution buffer. The entire volume of each nucleic acid extract was subjected to DNAse digestion with 0.25 U of TURBO DNAse (Thermo Fisher Scientific) in 30 μl reactions incubated for 30 min at 37°C. Digestion products were cleaned up using 2× AMPure XP Beads (Beckman Coulter) following the manufacturer’s instructions, with a final elution volume of 10 μl nuclease-free water. The 10 μl volume of DNAse-digested RNA extracts were used to generate DNA libraries with the KAPA RNA HyperPrep Kit (Roche). The manufacturer’s protocol recommends a minimum RNA input of 1 ng. As our inputs were substantially lower, we made several modifications to the protocol (Supplementary Table S1). For indexing, HT Dual Index Duplex Adapters (Integrated DNA Technologies) were used.

### QC Metrics for Library Prep and Target Enrichment

To establish the quality control (QC) metrics for the consistent generation of HCV-enriched DNA libraries, we quantify the relative amount of HCV fragments in the RNA extracts and their relative DNA libraries by HCV-specific qPCR assay using the QuantiTect Virus kit (Qiagen) and the KAPA Probe Fast Universal kit (Sigma Aldrich), respectively. Both qPCR assays were performed according to the manufacturer’s instructions using previously described primers and probes targeting HCV (Davalieva et al., 2014). Total mass of the libraries was determined using Quant-it dsDNA HS Assay Kit (Invitrogen), following the manufacturer’s specification.

### Sequence Capture

Each HCV target enrichment experiment carried out during the technical validation was performed using batches of 72 DNA libraries (which included two positive controls, four NHP controls, and four nuclease-free water controls). Considering the range of relative abundances of viral inserts in each individual DNA library determined by HCV-specific qPCR, each batch of DNA libraries were split into two groups: Low HCV cycle threshold (Ct) and High HCV Ct. The definition of “low” and “high” Ct was assessed individually per experiment. Depending on the range of Ct values of all libraries included in the experiment, a Ct value which allows the samples to be divided into two pools with: (i) a similar number of samples in each one; and (ii) the sample library with lowest Ct is 6 to 10 Ct cycles different from the library with higher Ct in the Low Ct pool and 10–20 in the High Ct Pool. DNA libraries assigned to each group were pooled by mass as determined in section QC metrics for library prep and target enrichment, adding the same number of nanograms of each DNA library to the pool. A total of 500 ng of each pool were used to perform the subsequent capture of HCV fragments.

The pooled DNA libraries were hybridized and captured using 120-nt HCV-specific biotinylated oligonucleotide probes targeted against genotypes 1–6 (Integrated DNA Technologies) and NimbleGen SeqCap target enrichment reagents (Roche) following manufacturer’s specifications, but instead of Multiplex Hybridization Enhancing Oligo Pool, using xGen Blocking Oligos (Integrated DNA technologies) (Bonsall et al., 2015). To each pooled DNA library, we added 6 pmol HCV-specific probes to the hybridization reaction, then incubated them in a thermocycler at 47°C (with the lid heated to 57°C) for 24 h. Following hybridization, the HCV DNA libraries bound to the biotinylated probes were partitioned using magnetic streptavidin-coated beads and the “low” and “high” Ct pools were subjected to a further 12 or 16 cycles of PCR amplification, respectively. The concentrations of both amplified pools (ng/μl) were determined as described above, and the two pools were themselves pooled, adding the same relative quantity of HCV-specific library per pool.

### Next Generation Sequencing

Prior to sequencing, the final pool was quantified using the KAPA SYBR FAST Universal qPCR Kit for Illumina libraries (KAPA Biosystems) on a 7500 Real-Time PCR System (Applied Biosystems), and analyzed for fragment size distribution using the High Sensitivity DNA Kit (Agilent) on a 2100 Bioanalyzer Instrument, both following manufacturers’ specifications. Sequencing was performed on an Illumina MiSeq instrument using the MiSeq Reagent Kit V2 (300 cycles) (Illumina), with the following minor modifications to the manufacturer’s guidelines. The final pool was diluted to 2 nM and denatured with 0.2 N sodium hydroxide for 2 min rather than five, incubated for 4 min at 95°C, and diluted in kit reagent HT1 to produce 1 ml of a 20 pM solution. These were further diluted to make 700 μl of a 9 pM solution, of which 10% was substituted with 12.5 pM PhiX sequencing control V3 (Illumina). A volume of 600 μl of this final solution was loaded onto the MiSeq cartridge.

### Amplicon-Based Sanger Sequencing

Viral RNA was extracted from 200 uL of plasma using QIAamp UltraSens Virus Kit (QIAGEN) and eluted in a final volume of 60 μL. A subtype-specific PCR assay was used to amplify sequences in NS5A gene (positions 6095 to 7833) from subtype 1a samples. Briefly, cDNA was generated using 5 μL of viral RNA extract and SuperScript II One-Step RT-PCR System with Platinum *Taq* DNA Polymerase (Invitrogen) followed by nested PCR using Platinum *Taq* DNA Polymerase (Invitrogen). Sanger sequencing used the dideoxy ABI sequencing systems in both directions using overlapping internal primers. Primers used for RT-PCR, nested PCR and sequencing, and cycling conditions are indicated in Supplementary Table S2. A previously described pan-genotypic PCR assay was used to amplify a fragment of the NS5B gene from samples of different genotypes and subtypes (Mellor et al., 1995). Sequences were analyzed using Sequencher software (Gene Codes) and aligned using subtype-specific consensus sequences.

Sequences obtained from each method were compared with those derived from NGS methods (global consensus), and the numbers of nucleotide and amino acid sequence differences were recorded.

### Bioinformatic Pipeline

De-multiplexed, adapter-stripped, paired end FASTQs were subjected to human read removal by competitive mapping with SMALT (downloaded from www.sanger.ac.uk/science/tools/smalt-0) against an index compiled from a combined FASTA set containing both the hg38 human genome reference and an HCV reference genome set comprising 164 subtype-annotated HCV whole genome sequences obtained from the Los Alamos sequence database (Kuiken et al., 2005). Reads mapping to the human reference at either end were discarded and surviving paired-end reads were reconstituted using samtools v1.8 (Li et al., 2009) and mapped to the HCV reference genome set, with the frequencies of hits to each subtype recorded using “Splitpops” tool.^1^

FASTQs were subjected to *de novo* assembly using VICUNA v1.3 (Yang et al., 2012). In cases where a complete genome was not assembled directly, LASTZ v1.02 (Harris, 2007) used the subgenomic contigs to select the best-matching reference from the HCV reference set, and attempted to iteratively close gaps between contigs with BWA MEM (Li, 2013). The final BAM file was processed using an in-house C++ program (QuasiBAM) (Penedos et al., 2015) to derive a consensus sequence, and a tabular file recording nucleotide frequency, depth and quality metrics for each nucleotide position in the mapping process. Positions with mixtures greater than 15% were coded as IUPAC ambiguities and those with depths of coverage below 30 were considered to be unreliable and thus coded as Ns. Regions containing Ns were not used in downstream analyses. Output data was then submitted to Geno2pheno[hcv] (Kalaghatgi et al., 2016) genotypic interpretation systems for assignment of genotype and interpretation of resistance.

### Generating Synthetic Quasispecies and FASTQ Datasets

An in-house script was used to generate synthetic quasispecies, expanding upon the approach described by Pandit and De Boer (2014). These were used to validate components of the bioinformatic pipeline. In brief, for a given HCV subtype, a multi-sequence alignment (MSA) of whole genome sequences was obtained from the Los Alamos HCV Sequence Database (Kuiken et al., 2005). Shannon entropy scores of the base frequency distributions for each column of the alignment were calculated to make a “weighted sites array.” Selection of genome loci to be mutated was by random sampling of this array, thus sites shown to be variable (albeit at the population level) were more frequently chosen than conserved regions. Furthermore, mutations at a locus were enacted by randomly selecting a sequence from MSA and substituting its base identity for the original, thus reinforcing conserved/variable regions. Indel variations were mutated *en bloc* rather than single sites.

A random sequence was selected from the MSA and by sampling the weighted sites array a number of times equal to 5% of the genome length and mutating these positions, a “parent” node sequence was generated. Child nodes were generated recursively, with the parental genome mutating between generations. The number of children per node was sampled from a normal distribution whose mean decreased as a function of distance from the parent node, thus limiting the number of generations and hence the size of the quasispecies. The output of this process is a hierarchical tree representing the relationships between each of the genome nodes, together with an MSA of all the constituent genomes. The median number of sequences per synthetic quasispecies was 1,579 (IQR 1,089-2,418).

To obtain quasispecies with specified characteristics, MSAs were further modified, either by introducing RAS into a selected percentage of lineage(s) (either linked or unlinked), by recombining two quasispecies at a specified breakpoint, and/or by randomly mixing quasispecies members at defined ratios to simulate dual infections.

Synthetic FASTQ datasets were prepared from synthetic quasispecies FASTA files using the open-source software tool ART (Huang et al., 2012). Mean and standard deviation insert size were specified from empirical data sets, and FASTA sampling parameters were set to maximize homogeneous representation of each input sequence. In-house scripts were used to mix synthetic FASTQ sets with FASTQ sets obtained from NHP libraries prepared as described above. To conceal their provenance, all reads in the combined datasets were shuffled and renamed prior to analysis.

## Results

### Establishment of Library Prep QC Metrics

DNA library preps made from RNA extracts of clinical samples from HCV-infected individuals with HCV Ct value <23 reproducibly resulted in more than 10% of total reads mapping to HCV with a genome coverage >90% at a read depth >30 after enrichment with HCV-specific probe baits. The median of the differential in HCV Ct values between the DNA library prep and respective RNA extract was 9.96 cycles [9.0–10.78; IQR].

### Sensitivity

**FIGURE 1:**
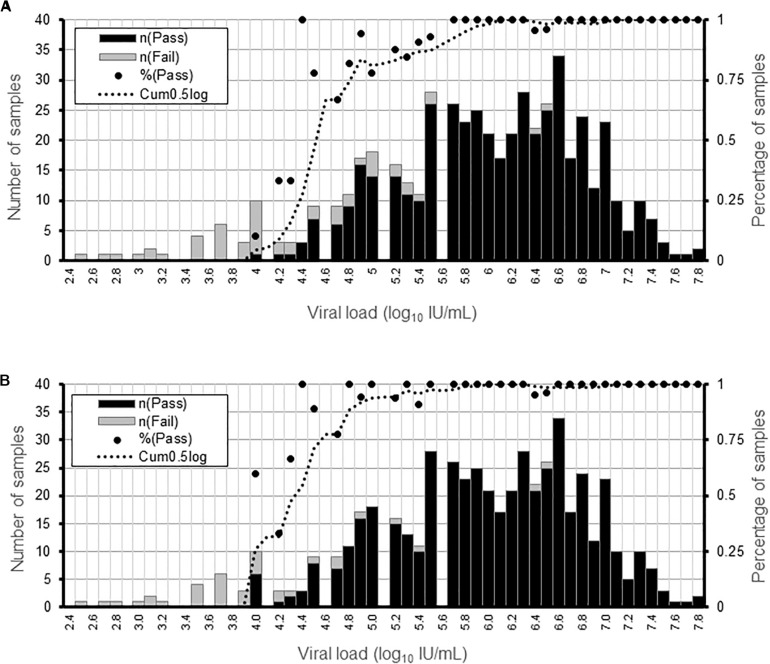
Determination of the sensitivity of the HCV WGS assay. Detection sensitivity was assessed as a function of viral load binned in 0.1 log10 IU/mL ranges. **(A)** Percentage of samples generating AVR data in NS3, NS5a, and NS5b genes at a minimum read depth of 30 in the different viral load ranges. **(B)** Percentage or samples generating genotyping data at a minimum read depth of 30 in the different viral load ranges. For each viral load range, the percentage of samples is shown by a black dot and the number of samples by the black and gray bars.

We determined the limit of detection for AVR analysis in NS3, NS5a, or NS5b genes and for genotyping using clinical samples containing HCV of different subtypes and viral loads. AVR data was generated in >90% and >50% of the samples at a viral load of ≥5.6 and ≥4.5 log_10_ IU/mL, respectively (Figure 1A). The limit of detection was lower for genotyping at ≥4.8 and ≥4.3 log_10_ IU/mL for >90 and >50% of the samples, respectively (Figure 1B).

### Accuracy

**FIGURE 2:**
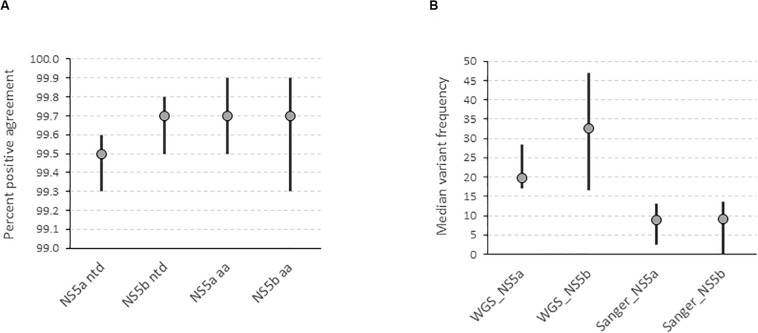
Determination of the accuracy of the HCV WGS assay. **(A)** Accuracy was assessed by determining the degree of agreement between the consensus FASTA sequences generated by the assay at a variant frequency threshold of 15% compared with the consensus FASTA sequence generated by the “gold standard” amplicon-based Sanger assay in NS5a and NS5b genes at nucleotide and amino acid level. **(B)** Determination of the variant frequency at discordant positions as determined by the WGS assay where the variant was called by either WGS or Sanger assay.

We determined the accuracy of the WGS assay by assessing the degree of agreement between the consensus sequences generated by the assay and the “gold standard” amplicon-based Sanger assay. This showed that the positive percent agreement (PPA) was 99.5% [99.3–99.6] at the nucleotide level and 99.7% [99.5–99.9] at the amino acid level for the NS5a region used for resistance-associated substitutions (RAS) testing in 17 subtype 1a samples (Figure 2A). Similarly, the PPA was 99.7% [99.5–99.8] at the nucleotide level and 99.7% [99.3–99.9] at the amino acid level in the NS5b region used for genotyping in 14 samples of varying subtypes (Figure 2A). The discordances were all due to mixed base sites where one method identified only one of two bases in a mixture. The exceptions were one instance where two of three bases were identified and one instance of complete discordance. In total, mixed bases were called at 216 of 10,863 positions (2.0%) and 39 of 4,606 positions (0.8%) in NS5a and NS5b, respectively. Of these, 157 (72.7%) in NS5a and 26 (66.7%) in NS5b were called by both methods. Of the total 72 mixed bases only called by one method, 39 were called by Sanger (54.2%) compared to 33 (45.8%) by WGS. The median frequency of variants called by WGS but not Sanger was 19.8% [17–28.4] and 32.5% [16.5–46.9] compared to 8.8% [2.5–13] and 9.1% [0–13.5] for variants called by Sanger but not WGS in NS5a and NS5b, respectively (Figure 2B and Supplementary Table S3). Most of the discordant mixtures (65/72; 90.3%) involved transition mutations (C↔T or G↔A). The nucleotide discordances resulted in 8 of 3,195 (0.25%) and 4 of 1,417 (0.28%) amino acid discordances in NS5a and NS5b, respectively.

### Inclusivity and Specificity

We investigated the ability of the assay to specifically detect genetic variations associated with AVR and the ability to distinguish the subtype of infecting virus. We used samples with known resistance markers and subtype that had been determined using the current “gold standard” Sanger-based sequencing, real-time PCR or probe-based assays. The genotyping sample panel consisted of 120 specimens, the majority (72.5%; *n* = 87) were of the most common subtypes in the United Kingdom, i.e., 1a, 1b, or 3a (Table 1). The remainder consisted of different “rare” subtypes (24.2%; *n* = 29) or samples where the subtype could not be determined by current methods (3.3%; *n* = 4). Overall, 116 of 120 samples, 96.7% [91.7–99.1], generated the same inferred genotype/subtype as the current methods. For the most common subtypes, the PPA between WGS and current methods was 98.9% [93.8–100]. In three of the four discordant results the “gold standard” methods assigned the wrong subtype whereas WGS identified the samples as being novel or unclassified subtypes (Table 2 and Supplementary Figure S1). In the remaining discordant sample the “gold standard” method was unable to assign a subtype whereas WGS did. In addition, there were three samples where the “gold standard” methods identified the correct genotype but were unable to assign a subtype and WGS confirmed these as novel or unclassified subtypes belonging to genotypes 1, 2, and 6.

**TABLE 1:** Subtype distribution and positive percent agreement for samples used for validation of HCV genotyping.

**Genotype**	**Subtype**	**Number of samples (% of total)**	**Proportion agreeing by WGS (%)**
1	a	41 (34.2)	100
	b	11 (9.2)	90.9
	l	2 (1.7)	100
	unclassified	2 (1.7)	50
2	a	2 (1.7)	100
	b	2 (1.7)	100
	j	1 (0.8)	0
	unclassified	1 (0.8)	100
3	a	35 (29.2)	100
	b	4 (3.3)	100
	c	1 (0.8)	0
	g	1 (0.8)	100
	k	1 (0.8)	100
4	a	1 (0.8)	100
	d	7 (5.8)	100
	k	2 (1.7)	100
	v	1 (0.8)	100
6	a	1 (0.8)	100
	f	1 (0.8)	100
	h	1 (0.8)	100
	r	1 (0.8)	100
	unclassified	1 (0.8)	100
Total		120 (100.0)	96.7

**TABLE 2:** Genotype and AVR of discordant samples.

**Sample no.**	**Genotyping**	
	**Gold standard assay (type)**	**WGS assay**
TV50	1-unknown subtype (NS5b sequencing)	1a
TV106	1b (NS5b sequencing)	1-novel subtype
TV5	2j (NS5b sequencing)	2-novel subtype
TV101	3c (line-probe)	3-novel subtype

**Sample no.**	**AVR**	
	**Gold standard assay (type)**	**WGS assay**

TV62	Y93CY (Sanger sequencing)	Y93 (C at 6% variant frequency)
TV51	M28AV (Sanger sequencing)	M28MV (V at 79% variant frequency)

For AVR, drug resistance markers were inferred from 21 samples which had a total of 26 RAS detected by Sanger sequencing. WGS could detect 24 of the 26 RAS, yielding a PPA of 92.3% [74.9–99.1]. The first RAS detected by Sanger sequencing but not WGS was a mixture Y93CY in NS5a (Table 2). Analysis of variant frequency from the WGS data showed that the Y93C variant was present but at a frequency of 6%, below the 15% variant frequency threshold set for WGS assay. The second discordant RAS was at position 28 in the NS5a gene of another sample where Sanger sequencing detected a mixture of A and V whereas WGS only identified V.

### Precision and Linearity

To assess the degree to which repeated sequencing of a specimen gave the same result, we performed intra-run and inter-run repeatability testing. For intra-run repeatability, for each sample three replicates of a DNA library were target-enriched separately and sequenced in the same MiSeq run. Inter-run repeatability was assayed firstly, by sequencing the same target-enriched DNA library prep in separate MiSeq runs and secondly, by generating replicate DNA libraries of the same specimen from different RNA extracts on different days (reproducibility). There was a strong positive correlation between median read depth of the replicates for both repeatability (*R*^2^ = 0.99) and reproducibility (*R*^2^ = 0.76) experiments (data not shown).

The mean genetic similarities for the intra- and inter-run repeatability experiments was >99.9% and exhibited very low measures of uncertainty with mean Shannon entropy <0.00017 (Table 3). Discordances were observed at ≤4 sites and were due to sites where a mixture was detected in one run with only one of the mixed bases present in the replicate or vice versa. The genetic similarities for the reproducibility experiments were again very high at >99.6%. However, the number of discordant sites was slightly higher ranging from 0 to 63 sites, as was the measure of uncertainty with mean Shannon entropy of 0.00178 (Table 3).

**TABLE 3:** Determination of assay precision and linearity.

**Description**	**Sample number (subtype)**	**Viral load (IU/mL)**	**Similarity (%)^*c*^**	**Number of discordant sites^*d*^**	**Mean Shannon entropy^*e*^**	**% positions with nucleotide frequency differences below**		
						**1%**	**2%**	**5%**
Intra-run repeatability^*a*^	TV22 (1a)	1,790,000	99.98	3	0.000211	93.81	99.43	99.91
	TV20 (1b)	2,470,000	99.99	1	0.00007	93.57	98.61	99.27
	TV33 (1a)	106,000	100	0	0	86.61	98.28	99.95
	TV32 (1a)	301,000	99.98	4	0.000282	87.65	97.19	99.64
	TV37 (3a)	425,000	99.98	4	0.000281	97.61	99.51	99.94
	Mean	–	99.97	2.4	0.000169	91.85	98.6	99.74
Inter-run repeatability^*b*^	TV33 (1a)	106,000	100	0	0	97.71	99.9	99.98
	TV22 (1a)	1,790,000	100	0	0	98.6	99.93	100
	TV20 (1b)	2,470,000	100	0	0	95.43	99.91	100
	TV32 (1a)	301,000	99.99	2	0.000155	94.98	99.74	100
	TV37 (3a)	425,000	100	0	0	98.75	99.97	100
	Mean	–	100	0.4	0.000031	97.09	99.89	100
Reproducibility^*b*^	TV65 (1a)	910,000	99.93	13	0.000997	88.7	95.82	98.84
	TV58 (1a)	995,000	99.96	8	0.000613	92.69	97.67	99.62
	TV48 (1a)	664,000	99.76	44	0.003375	81.4	90.72	97.54
	TV56 (1a)	3,340,000	99.8	37	0.002838	91.96	96.95	99.29
	TV46 (1a)	98,200	99.98	3	0.00023	81.13	89.36	96.8
	TV78 (1b)	63,700	100	0	0	94.24	98.74	99.9
	TV83 (1b)	857,000	99.65	63	0.004833	88.92	94.98	98.86
	TV74 (3a)	150,000	99.99	1	0.000076	93.67	98.47	99.93
	TV77 (3a)	111,000	99.78	40	0.003056	86.49	94.69	98.92
	Mean	–	99.87	23.2	0.00178	88.8	95.27	98.86

*^*a*^Calculated using three replicates. ^*b*^Calculated using two replicates. ^*c*^At nucleotide level and discordant sites were scored as 50% mismatch. ^*d*^All discordant sites consisted of the presence of a mixture in one sequence but only one of the bases present in the replicate e.g., Y in one sequence and C or T in the replicate. There were no complete mismatches. ^*e*^Shannon entropy was calculated using the Entropy-One tool (https://www.hiv.lanl.gov/content/sequence/ENTROPY/entropy_one.html).*

Next, we determined the consistency of detecting variant frequencies in repeatability and reproducibility experiments. The proportion of positions at which the difference in observed variant frequency of the sequence replicates at all nucleotides in the coding region of the HCV genome was <1% ranged from 88.8 to 97.09% for reproducibility and inter-run repeatability experiments, respectively. The proportion of positions approached 100% when the difference in variant frequency was <5%, ranging from 98.86 to 100% for reproducibility and inter-run repeatability experiments, respectively (Table 3). Regions with large differences in variant frequencies between replicates were distributed throughout the whole genome but were mostly associated with regions of high diversity or homopolymers, such as the E1/E2 region (Supplementary Figure S2).

**FIGURE 3:**
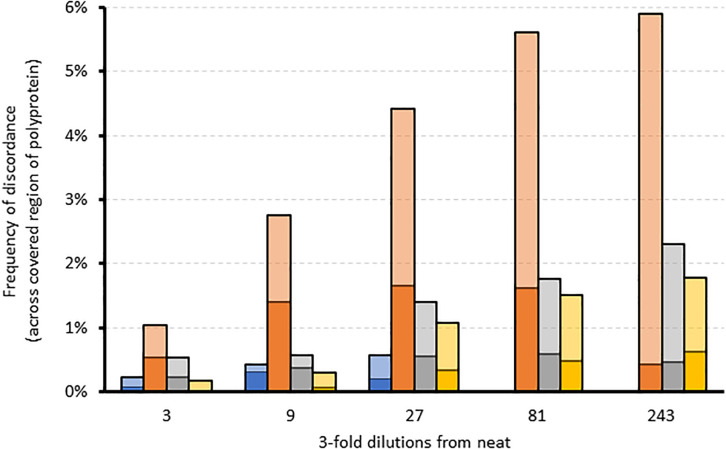
The effect of sampling bias on variant frequency detection using serial dilutions of different samples. Stacked bar graph comparing the frequency of amino acid (dark colors) and corresponding nucleotide (light colors) discordances across the region covered by each dilution relative to the corresponding neat sample. Blue = sample 1 (gt4a); Orange = sample 2 (gt2b); Gray = sample 3 (gt1a); Yellow = sample 4 (gt3a).

We further assessed the linearity of the assay by determining the effect of sampling bias on variant frequency detection. Variant frequencies were analyzed across the whole genome and specifically at drug resistance positions in serially diluted clinical samples of a variety of subtypes. Five three-fold dilutions were generated for four samples of subtype 1a, 2b, 3a, and 4a. As expected, overall genome coverage was 85–100% for the neat and first three dilution series but subsequently decreased across the dilution series (Supplementary Figure S3). We observed that the proportion of nucleotide positions discordant with the corresponding neat sample increased across the dilution series (Figure 3). In contrast, the proportion of amino acid discordances initially increased but eventually decreased (Figure 3). When analysis was limited to the 39 resistance-associated positions in NS3, NS5A, and NS5B, only a single discordance was observed between the dilution series and their corresponding neat sample. Position 31 in NS5A was mixed (M and L) in all but the final dilution of sample 2, where only M was detected. The frequency of M at this position varied across the dilutions from 71.6% in the neat sample, through 58.7, 61.7, and 37.6% in the first three dilutions with read depths of 493, 3885, 860, and 668, respectively. The position was not covered in the fourth dilution, and was present at 100% in the final dilution, at a depth of 2085.

**FIGURE 4:**
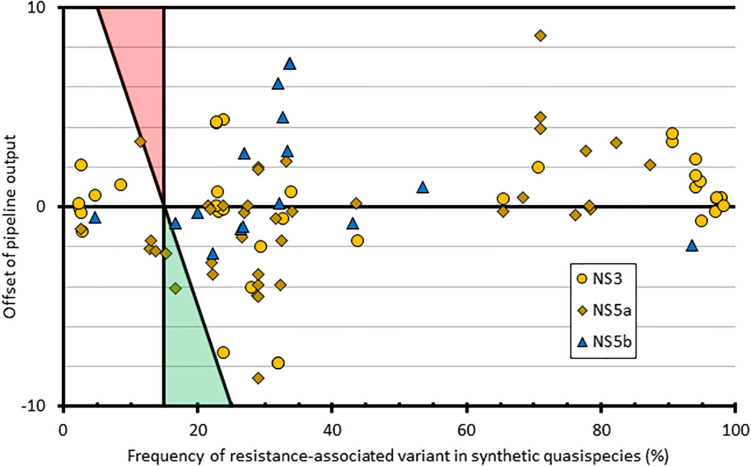
Graphical representation of how the pipeline outputs compare to quasispecies input at resistant loci with mixed amino acid frequencies. The *x*-axis comprises the frequency of each variant within the input quasispecies; the *y*-axis shows the difference between the input value and the pipeline output frequency at that variant. Shaded triangle regions represent the area where false positives (red) and false negatives (green) would be observed, using the pipeline variant calling threshold of 15%.

To assess the contribution of the bioinformatics pipeline to the variability in variant frequency calling, we used simulated FASTQ datasets generated from synthetic HCV quasispecies with different RAS introduced at predetermined but varying frequencies ranging from 1 to 100%. We then analyzed all the positions where the RAS variant was present at <100% (*n* = 94) and compared the bioinformatics pipeline outputs to the expected results (Figure 4). This showed variation in variant frequency calling of up to 8.6% above or below the expected value resulting in two missed calls of mutations present at a frequency greater than 15%. Both were of NS5a RAS in the same subtype 3a quasispecies: A30K at 15.2% was reported at 12.9%, and Y93H at 16.6% was reported at 12.5%.

### Post-sequencing Quality Control Checks

One of the biggest quality assurance challenges of WGS is cross-contamination that can occur during the laboratory processing of the samples, a phenomenon identified as a major issue during the assay development phase (Thomson et al., 2016). We used simulated FASTQ datasets generated from synthetic HCV quasispecies and sequencing outputs from NHP libraries mentioned above to determine the ability of the bioinformatics pipeline to detect and distinguish between cross-contaminants, mixed infections and recombinant viruses because no “gold standard” reference standards currently exist.

Analysis of each synthetic subtype quasispecies datasets by the Splitpops software of the bioinformatics pipeline yielded the correct identification of a primary majority subtype population. However, the pipeline also reported a secondary minority population at <5% belonging to a different subtype but same genotype as the majority population e.g., a 1c minority population reported for a quasispecies simulated from a 1a population (Supplementary Table S4A).

For mixed infection datasets, the ratios and subtypes of the constituent populations were correctly estimated by the Splitpops software down to approximately the 20:1 ratio, thus representing a secondary population at ≥5% (Supplementary Table S4B). Analysis of the recombinant virus datasets also resulted in detection of the different subtypes constituting the recombinant virus if the secondary subtype of the recombinant strain was present at ≥9.6%; however, the Splitpops software was less accurate at determining the exact ratios of constituent subtypes making up the recombinant virus (Supplementary Table S4B). When the synthetic datasets were mixed with background reads containing human pegivirus, a virus distantly related to HCV and associated with HCV infections (Bonsall et al., 2016; Wang et al., 2018; Sridhar et al., 2019), Splitpops could identify the primary infection in single infections and both subtypes in mixed infections (Supplementary Table S4C). However, the percentage of mapped reads belonging to HCV was always significantly lower than expected (<82%); however, this was relative to the percentage of background reads added and did not affect the expected ratio of subtypes in the mixed infections.

**FIGURE 5:**
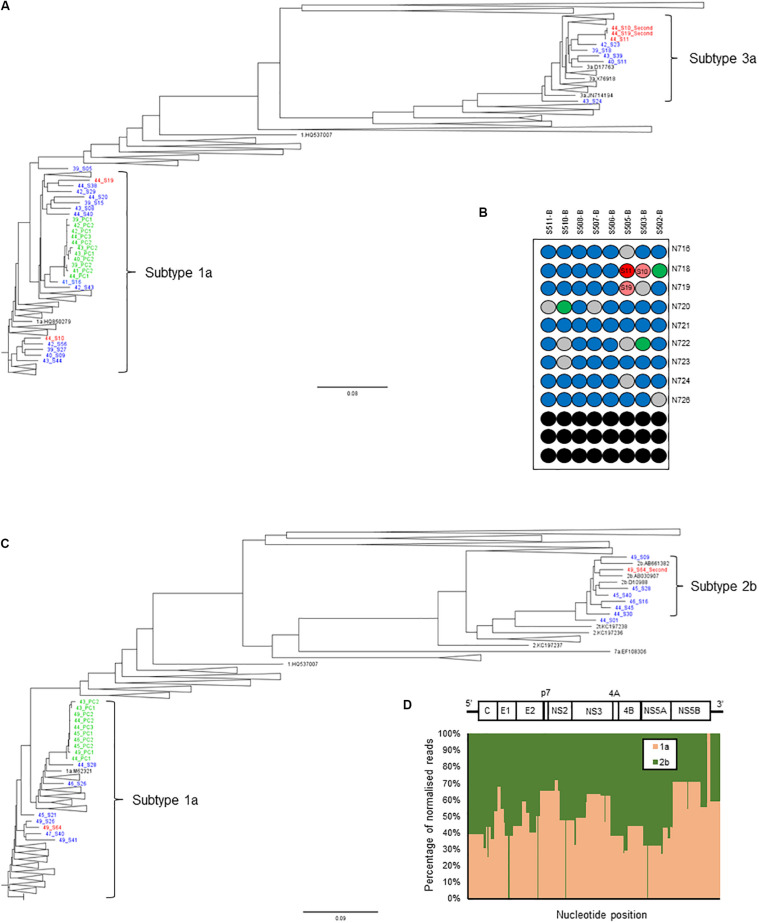
Additional bioinformatics analyses undertaken on samples with Splitpops outputs that did not satisfy established primary and secondary population thresholds. **(A)** Phylogenetic reconstruction of NS5B sequences showing a cross-contamination event. Three samples: 44-S10, 44-S11, and 44-S19 (red font) in experiment no. 44 were involved and are analyzed together with sequences from five previous experiments, nos. 39-43 (blue font). Green font = positive controls and black font = subtype references. **(B)** Position of samples involved in the cross-contamination event are mapped on the library prep 96-well plate for experiment no. 44. Sample 44-S11, the source of contamination (dark red) and contaminated samples: 44-S10 and 44-S19 (light red) are indicated. Blue = other samples, gray = negative controls, green = positive controls and black = blank wells. The pair of adapter indexes used are shown adjacent to each row. **(C)** Phylogenetic reconstruction of NS5B sequences showing a mixed infection. Sample 49-S64 (red font) in experiment no. 49 was a mixed infection (subtypes 1a and 2b) and is analyzed together with sequences from five previous experiments, nos. 43-48 to rule out cross-contamination. Green font = positive controls and black font = subtype references. **(D)** Graphical representation of the distribution of the reads in sample 49-S64 by subtype shown as a percentage (*y*-axis) across the HCV genome (*x*-axis). The HCV genome is shown on top of the graph for reference.

Thus, we set a Splitpops threshold of >5% for the secondary population and/or <85% for the primary population to flag the presence of mixed infection, recombinant virus, unclassified subtypes or cross-contamination in clinical samples. This finding triggers the performance of additional bioinformatics analyses to rule out cross-contamination and confirm any finding of mixed infection or recombinant virus. This involved: (i) phylogenetic reconstruction from individual HCV gene regions of all sequences in the MiSeq run and previous five runs; (ii) genome assembly from reads of the separate populations to determine if full-length genomes are present; and (iii) similarity plot analysis of the consensus whole genomes to determine possible recombination break points or unclassified subtypes. Table 4 shows examples of Splitpops outputs from experiments with samples that have a secondary population at >5% and/or a primary population at <85%. For cross-contamination, the phylogenetic reconstruction of samples 44-S10 and 44-S19 showed that the two samples with a secondary population at >5% contained contaminating reads from another sample in the same experiment, 44-S11. Both 44-S10 and 44-S19 were subtype 1a and the contaminant sample 44-S11 was subtype 3a. The reconstructed subtype 3a sequence from 44-S10 and 44-S19 were closely related and clustered with the sequence from 44-S11 in the NS5b phylogenetic tree (Figure 5A) and the other HCV domain phylogenetic trees (data not shown). The three samples were in wells next to each other in the same or adjacent column of the library prep 96-well plate for experiment no. 44 (Figure 5B).

**TABLE 4:** Summary of Splitpops module output showing representative examples of different clinical sample scenarios.

**Scenario**	**Sample ID**	**Reads**		**% mapped reads^c^**	**Splitpops 1 subtype**	**% mapped reads**	**Splitpops 2 subtype**	**% mapped reads**
		**Trimmed^a^**	**Filtered^b^**					
Single infection	44-S11	197,162	196,010	100	3a	99.7	other	0.2
	44-PC2	23,918	23,506	100	1a	91.7	1l	2.7
Contamination	44-S19	20,160	4,641	93.76	1a	67.5	3a	22.9
	44-S10	19,529	18,977	99.19	1a	84.8	3a	6.3
Mixed infection	49-S64	25,566	25,454	100	2b	56.3	1a	39.3
	59-S5	43,225	39,630	98.37	3a	81.3	1a	15.4
Recombinant virus	10-S43	22,798	21,754	96.29	1b	58.0	2k	33.9
	41-S22	254,285	253,624	99.84	1a	71.0	4o	10.5
Unclassified subtype	33-S10	174,341	160,437	99.88	1c	34.5	1b	15.5
	41-S10	48,461	47,621	93.56	2q	26.6	2	21.8

*^*a*^Number of reads after removal of adapters and low quality sequences.^*b*^Number of reads after human read removal by competitive mapping against FASTA set containing the hg38 human genome reference dataset. ^*c*^Percent of filtered reads that mapped to HCV reference dataset.*

**FIGURE 6:**
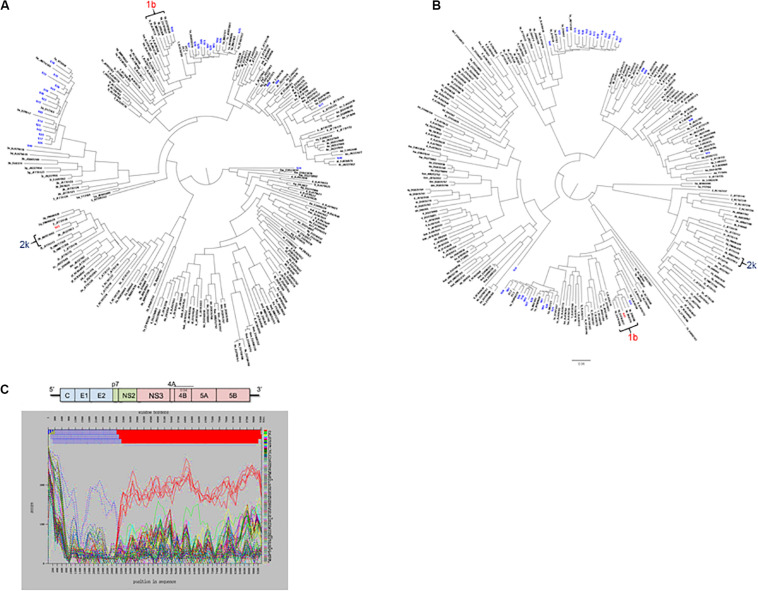
Additional bioinformatics analyses undertaken on samples with Splitpops outputs that did not satisfy established primary and secondary population thresholds. Phylogenetic reconstruction using core **(A)** and NS5B **(B)** gene sequences from experiment no. 10 showing clustering of sample S43 with subtype 2k and 1b reference sequences, respectively. Blue font = other sequences from experiment no. 10 and black font = subtype references. **(C)** Graphical representation of a similarity plot of S43 sequence showing its similarity to subtype 2k in the 5′-end structural genome region (blue) and to subtype 1b in the 3′-end non-structural genome region (red). The HCV genome map is shown on top of the graph for reference.

For mixed infection, two sequences were assembled from sample 49-S64 (subtypes 2b and 1a) that were independently located on the NS5b phylogenetic tree (Figure 5C) and were not closely related to other sequences in the same or the five previous runs. Further bioinformatic analysis showed a uniform distribution of subtypes 2b and 1a across the whole genome at proportions equivalent to those determined by Splitpops (Figure 5D). This confirmed the sample as a mixed infection. Lastly, with regards recombinant virus only one whole genome sequence was assembled from sample 10-S43. Phylogenetic analyses showed that the sequence clustered with subtype 2k sequences in the core gene (Figure 6A) and other 5′-end structural gene regions: 5′-UTR, E1 and E2 (data not shown) but clustered with subtype 1b in NS5b gene (Figure 6B) and other 3′-end non-structural gene regions: NS3, NS4b, and NS5a (data not shown). Similarity plot analysis confirmed the sequence to be a recombinant 2k/1b with a breakpoint between structural and non-structural gene regions (Figure 6C).

## Discussion

We performed the technical validation of a genotype-agnostic HCV WGS assay for the simultaneous detection of genotype and AVR in the clinical pathway. An assessment of the analytical performance characteristics of the assay showed comparable and often superior results compared to current “gold standard” assays. One exception was sensitivity, defined as the lowest concentration of HCV in a clinical sample that generated reportable sequence data in the clinically relevant regions. This was determined to be approximately 4.3 log_10_ IU/mL for genotyping and 4.5 log_10_ IU/mL for AVR for the HCV WGS assay. As expected, this is higher than that of PCR-based sequencing assays which can generate positive results on samples with viral loads of 3.0 log_10_ IU/mL or less because they employ gene-specific primers and significantly more cycles of amplification. However, 4.3 and 4.5 log_10_ IU/mL are considerably below the viral load of most blood samples from treatment-naïve HCV-infected patients we routinely process which is usually over tens of thousands IU/mL.

On the other hand, the WGS assay showed high specificity and achieved a PPA of >99.6% at nucleotide level and ≥99.9% at the amino acid level in the NS5a and NS5b genes of different genotypes and subtypes compared to the current “gold standard” Sanger sequencing assay. The few observed discordances were due to mixed base positions where only one base of the mixture was detected in one of the assays but with no specific trend. A minority of the nucleotide discordances resulted in amino acid changes (12/66, 18.2%), suggesting that they often occur at synonymous sites where underlying quasispecies variation is present and thus may be a result of stochastic sampling rather than assay polymerase errors. This was further confirmed by repeatability and reproducibility experiments where more variation in variant frequency calls was observed for the latter with the few differences again located at mixed base positions. On average, 2 and 23 discordances were observed over the whole genome for repeatability and reproducibility experiments, respectively. However, the variant frequency differences between duplicates were still very small, at ∼2% compared to ∼1% at the 95th percentile for reproducibility and repeatability experiments, respectively, with the large differences in variant frequencies mostly associated with regions of intrinsic high genetic diversity. These data were further supported by high similarities in sequence data generated by both experiments which was >99.6%. However, precision experiments on variant frequency detection using serial dilutions suggest sampling bias does significantly affect the efficiency of detection of minor variants and could thus be highly variable at low viral load.

Most importantly, the PPA for assignment of virus subtype was 98.9% for the most common circulating subtypes in the United Kingdom (subtypes 1a, 1b, and 3a) and 96.7% overall compared to the current “gold standard” genotyping assays. Discordances were either due to the WGS assay being able to assign a subtype where the current genotyping couldn’t or where current genotyping methods assigned wrong subtypes to novel unclassified subtypes. In addition, the WGS assay identified novel subtypes where the current assays could only assign genotype. Thus, WGS is more accurate at assigning the subtype of infecting virus compared to the current “gold standard” methods. Similarly, the WGS assay had a PPA of 92.6% for detection of AVR markers compared to Sanger sequencing assays. This was assessed over 26 markers in NS3, NS5a or NS5b of subtype 1a viruses. The two discordant results were due to the presence of mixtures. In the first discordant sample, the Y93CY mixture in NS5a gene was detected by the Sanger assay, which uses a variant frequency threshold of 20%. However, the WGS assay detected the Y93C variant at 6%, which is below the 15% variant threshold used by the WGS assay and is recommended for interpretation of NGS data (Zeuzem et al., 2017; European Association for the Study of the Liver, 2018; Panel, 2018; Bradshaw et al., 2019). We surmise that the Sanger assay inaccurately detected this variant to be present at >20% as it estimates variant frequencies from the height of chromatogram peaks whereas the NGS method used by the WGS assay is based on clonal sequencing and quantifies variant frequencies from individual reads which is more accurate. A study comparing the accuracy of Sanger sequencing at 20% variant frequency threshold to NGS at 20% and 15% variant frequency thresholds showed a high level of agreement at 99.6% [96.1–100%; range] and 99.4% [88.5–100%; range], respectively (Parkin et al., 2020). For the second discordant sample, the WGS assay detected the mixture M28MV in the NS5a gene whereas the Sanger assay detected the mixture M28AV. The M28MV mixture in WGS assay was represented by two codons: ATG = M (21%) and GTG = V (79%). The codon mixture in the Sanger assay was GYG giving the two possible codons GTG = V and GCG = A. It is likely that the G/A mixture at the first position was not detected by the Sanger method as it is around the 20% variant frequency threshold whereas the C/T mixture at the second position is a transition mutation which is one of the most frequent PCR generated errors. Sanger sequencing assays, which use gene-specific primers and significantly more PCR cycles, are more likely to introduce errors and sampling bias which may skew the exact variant frequency in a sample. A recent study comparing sequence capture and amplicon-based NGS methods has shown that the latter detects more low-frequency variants than the former (Mbisa et al., 2020). This is likely to be a result of an overcall of low-frequency variants by amplicon-based methods rather than a lack of sensitivity by sequence capture. However, this requires further investigation using standard reference material with well-characterized frequencies at mixed base positions.

Accuracy experiments provided further evidence for this phenomenon. We observed high agreement at both nucleotide and amino acid levels between WGS and Sanger (≥99.5%). The discordances were mainly due to mixed bases where one method called only one of the bases in a mixture with the Sanger method slightly overcalling mixed bases compared to WGS (39 vs. 33). Interestingly, the median variant frequency at positions where mixed bases were called by WGS but not Sanger was close to the 20% threshold of Sanger technology (19.8 and 32.5% for NS5a and NS5b, respectively) whereas it was significantly lower for the reverse scenario (8.8 and 9.1% for NS5a and NS5b, respectively). This could be a result of PCR errors and sampling bias of the Sanger method compared to the WGS method. Most of the mixed bases involved transitions C/T (Y) or A/G (R), the most common PCR errors most of which did not result in amino acid changes and thus had minimal impact on test accuracy which was >99.5%. Again, these data show that the WGS assay is more accurate and thus superior at detection of specific AVR markers and for determination of consensus sequence composition compared to amplicon-based Sanger sequencing assays.

One of the most significant quality assurance challenges for the sequence capture HCV WGS assay, and NGS assays in general, is cross-contamination from sequences within the same run which can occur during sample handling or carryover contamination as well as artifacts introduced by the Sequencer (Nelson et al., 2014; Sehn et al., 2015; Seitz et al., 2015; Thomson et al., 2016). On the other hand, it has been reported that mixed infections and recombinant viruses can occur at high frequencies depending on the characteristics of the infected population (Mcnaughton et al., 2014, 2018; Susser et al., 2017). Thus, in addition to implementing stringent laboratory measures like unidirectional molecular workflows and robust MiSeq maintenance washing and procedures, it is critical to implement a bioinformatics solution that can accurately detect mixed infections or recombinant viruses and distinguish these incidences from cross-contamination (Lee et al., 2016). This is because the former may require different therapeutic approaches (Mcnaughton et al., 2014; Hedskog et al., 2015) whereas the latter may result in reporting of the wrong result. To address this quality assurance challenge, we used outputs from the Splitpops software to determine thresholds for detection of the different scenarios. Splitpops separates HCV reads generated from the MiSeq run into respective HCV subtypes using a reference sequence database according to genetic similarity. We validated this functionality using simulated datasets of HCV that mimicked mixed infections of different subtypes at different frequencies and recombinant viruses with different breakpoints observed in clinical samples. We then compared outcomes from Splitpops to the expected results, an exercise that is not possible using clinical samples with unknown composition. Splitpops usually generates a secondary HCV subtype population that constitutes <5% of total HCV reads belonging to the same genotype as the primary population. This is due to similarities of HCV genomes in 5′ and 3′ UTR regions within genotypes and rarely across genotypes. Thus, the presence of a secondary population within the same genotype as the primary population present at >90% indicates a single subtype infection. For samples where a secondary population is detected at >5%, further bioinformatics analyses are then performed to distinguish the origins of two or more populations. We also show that the presence of background human pegivirus reads can result in proportional reduction in the percentage of total filtered reads mapping to HCV. Thus, a threshold of <85% for the primary Splitpops population should also trigger additional bioinformatics analysis to confirm the absence of mixed infection or recombinant virus as the percentage of the secondary population would be reduced. Using clinical samples, we are able to show that this is an effective quality assurance approach. Future quality improvements to the assay would include: (i) the inclusion of probes targeting genotype 7 and 8 even though they are not genotypes commonly circulating in the United Kingdom, and (ii) the use of HCV-GLUE, a recently developed software for interpretation of genotypic resistance. A significant advantage of HCV-GLUE is that it can process individual reads submitted as BAM files thereby providing accurate identification of amino acid frequencies and has a well-curated resistance data schema. It thus represents the most appropriate approach for interpreting viral deep sequencing data (Singer et al., 2018, 2019). The software is also regularly updated with emerging *in vivo* and *in vitro* evidence on HCV drug resistance.

To summarize, we have developed an HCV WGS assay for the simultaneous detection of genotype and AVR to be used for direct patient care and performed the technical validation of the assay’s analytical performance. The assay has several advantages over current PCR-based Sanger sequencing or NGS assays which include accurate genotyping, detection of mixed infections, accurate detection of low frequency drug-resistance variants, high-throughput and theoretically reduced costs. To our knowledge, this is the first report of an end-to-end technical validation of a viral WGS assay, and provides a benchmark for clinical microbiology laboratories implementing viral NGS assays as the technology gradually replaces Sanger sequencing.

## Data Availability Statement

The datasets presented in this article are not readily available because they contain identifiable human genome sequences. Requests to access the datasets should be directed to JM (tamyo.mbisa@phe.gov.uk).

## Ethics Statement

All experiments were performed in accordance with the ‘Guidance on Conducting Research in Public Health England’ (Version 3, October 2015; Document code RD001A). This study only involved the use of archived, residual samples that were sent to the National Reference Laboratory for routine diagnosis and sequence characterization with consent for leftover sample to be used in other assays. The samples were anonymized by removal of any patient identifiable information and assignment of a non-specific project code.

## Author Contributions

CM, DFB, RB, EB, DB, and JM conceived of the project. CM, HM, RP, and YC performed the laboratory experiments. CM, DFB, KL, and JM performed data curation and analysis. DFB, KL, RM, DW, RB, MA, and CI provided resources for the project. CM, DFB, and JM wrote the original draft of the manuscript. CM, DFB, MA, CI, RM, DW, EB, DB, and JM reviewed and edited the manuscript. RM, RB, EB, and JM secured funding for the project. All authors have read and approved the manuscript.

## Conflict of Interest

The authors declare that the research was conducted in the absence of any commercial or financial relationships that could be construed as a potential conflict of interest.

## Footnotes

1 https://github.com/camilla-ip/AnalysisTools

## Supplementary Material

Supplementary Figure S1 Phylogenetic reconstruction of using WGS data of samples used for genotyping technical validation. The polyprotein coding sequences of the 120 clinical samples representing the subtype diversity in the United Kingdom were aligned together with 166 HCV reference sequences from the Los Alamos HCV database. The MSA was used for reconstruction of a maximum-likelihood tree using FastTree software under GTR nucleotide substitution model. Green branches = clinical sample sequences where subtype assignment was concordant between WGS and “gold standard” methods; red branches = clinical sample sequences where subtype assignment was discordant between WGS and “gold standard” methods; blue branches = clinical sample sequences where WGS classified it as “novel” subtype but “gold standard” methods only identified the genotype but not the subtype; black branches = reference sequences.

Supplementary Figure S2 Variant frequency variability between replicates of patient samples. Bars represent the largest difference in observed variant frequency along the HCV genome of sample-specific reference sequence between reproducibility–interrun–replicates (panel **A**) and repeatability–intrarun–replicates (panel **B**) at minimum read depth of 30. Reproducibility experiments were performed as duplicates whereas repeatability experiments as triplicates.

Supplementary Figure S3 Genome coverage by serial dilution of clinical samples used for linearity experiments. Bars indicate HCV polyprotein coverage across neat (1) and four three-fold dilutions (2, 3, 4, and 5) of four clinical samples. Blue = sample #1, orange = sample #2, gray = sample #3 and yellow = sample #4.

Supplementary Table S1 List of modifications made to the manufacturer’s protocol when using the KAPA RNA HyperPrep Kit to construct libraries from low RNA inputs.

Supplementary Table S2 Details of NS5A amplicon-based Sanger sequencing assay.

Supplementary Table S3 Variant frequency at WGS and Sanger discordant mixed base positions.

Supplementary Table S4A Comparison of expected and Splitpops outputs for pure subtypes synthetic data.

Supplementary Table S4B Comparison of expected and Splitpops outputs for mixed infection and recombinant virus synthetic data.

Supplementary Table S4C Comparison of expected and Splitpops outputs for pure subtype or mixed infections synthetic data combined with NHP background reads also containing human pegivirus.
