# Supplementary figures and images for: Technical Validation of a Hepatitis C Virus Whole Genome Sequencing Assay for Detection of Genotype and Antiviral Resistance in the Clinical Pathway
Source: Front Microbiol. 2020 Oct 9;11:576572. doi: 10.3389/fmicb.2020.576572 (PMC7583327; doi:10.3389/fmicb.2020.576572)

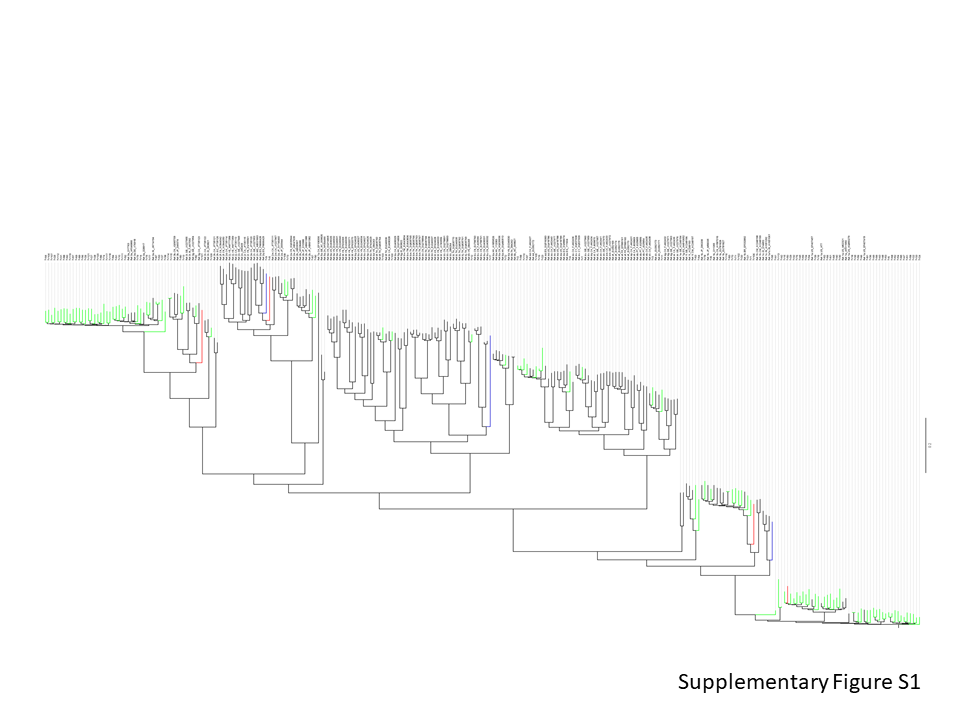

Supplement: Supplementary Figure S1 — Phylogenetic reconstruction of using WGS data of samples used for genotyping technical validation. The polyprotein coding sequences of the 120 clinical samples representing the subtype diversity in the United Kingdom were aligned together with 166 HCV reference sequences from the Los Alamos HCV database. The MSA was used for reconstruction of a maximum-likelihood tree using FastTree software under GTR nucleotide substitution model. Green branches = clinical sample sequences where subtype assignment was concordant between WGS and “gold standard” methods; red branches = clinical sample sequences where subtype assignment was discordant between WGS and “gold standard” methods; blue branches = clinical sample sequences where WGS classified it as “novel” subtype but “gold standard” methods only identified the genotype but not the subtype; black branches = reference sequences. [file Image_1.TIF]

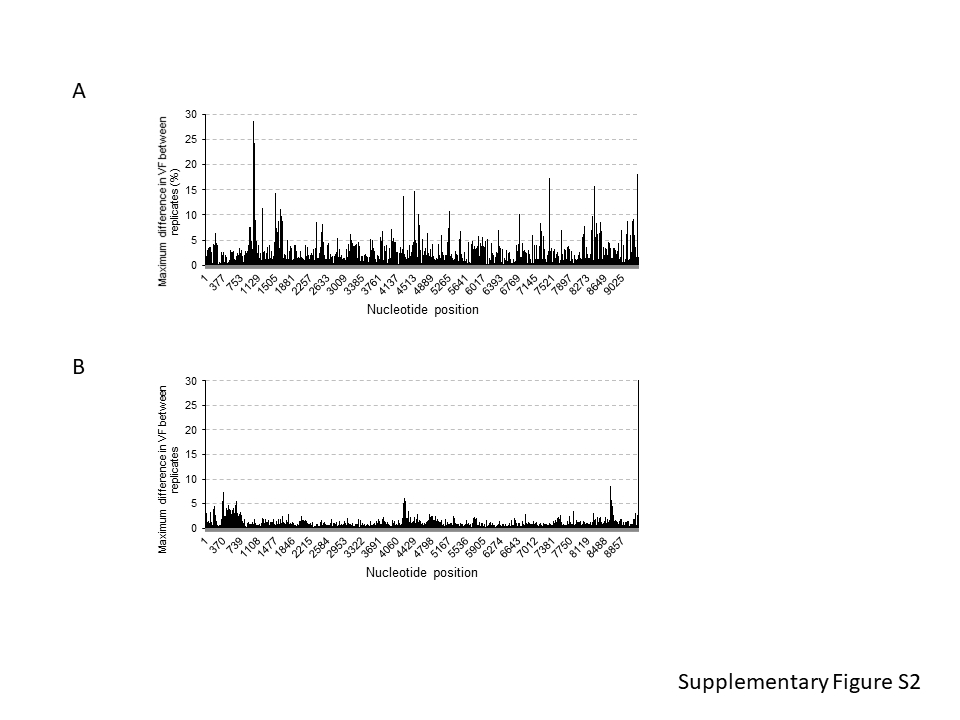

Supplement: Supplementary Figure S2 — Variant frequency variability between replicates of patient samples. Bars represent the largest difference in observed variant frequency along the HCV genome of sample-specific reference sequence between reproducibility–interrun–replicates (panel A) and repeatability–intrarun–replicates (panel B) at minimum read depth of 30. Reproducibility experiments were performed as duplicates whereas repeatability experiments as triplicates. [file Image_2.TIF]

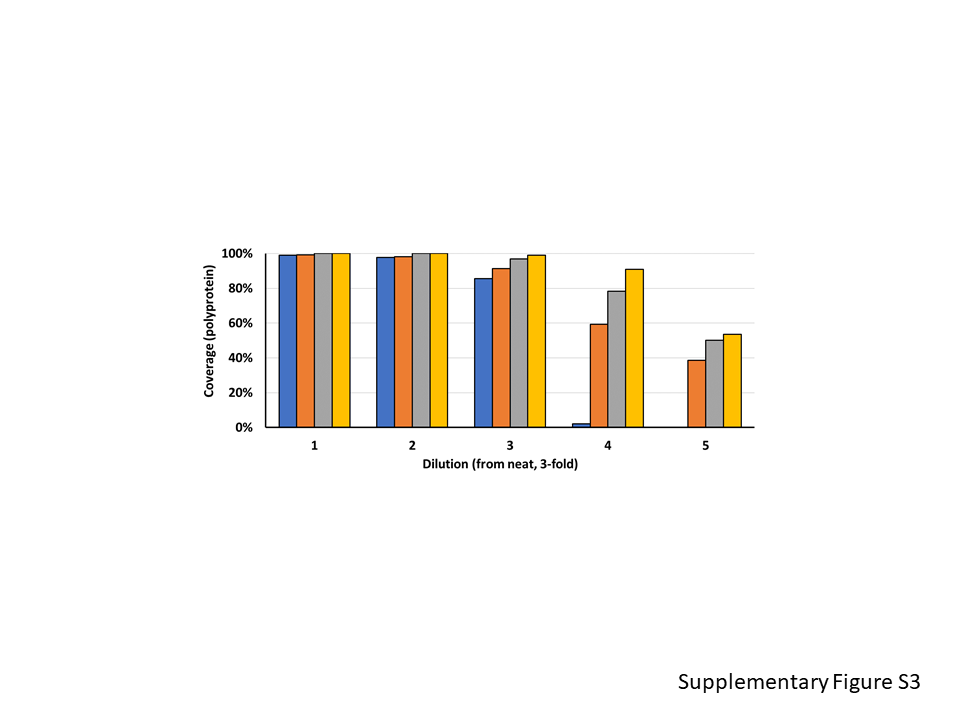

Supplement: Supplementary Figure S3 — Genome coverage by serial dilution of clinical samples used for linearity experiments. Bars indicate HCV polyprotein coverage across neat (1) and four three-fold dilutions (2, 3, 4, and 5) of four clinical samples. Blue = sample #1, orange = sample #2, gray = sample #3 and yellow = sample #4. [file Image_3.TIF]
