# Supplementary material for: Technical Validation of a Hepatitis C Virus Whole Genome Sequencing Assay for Detection of Genotype and Antiviral Resistance in the Clinical Pathway
Source: Front Microbiol. 2020 Oct 9;11:576572. doi: 10.3389/fmicb.2020.576572 (PMC7583327; doi:10.3389/fmicb.2020.576572)
Supplement: Supplementary Table S1 — List of modifications made to the manufacturer’s protocol when using the KAPA RNA HyperPrep Kit to construct libraries from low RNA inputs. [file Table_1.DOCX]

**Supplementary Table S1:** List of modifications made to the manufacturer’s protocol when using the KAPA RNA HyperPrep Kit to construct libraries from low RNA inputs.

| **STEP** | **Manufacturer’s protocol** | **Modified protocol** |
| --- | --- | --- |
| RNA fragmentation | **Final reaction volume: 20µl**   - 10µl RNA - 10µl “Fragment, Prime and Elute Buffer (2X)”   Incubate at 94°C for 6-8 min | **Final reaction volume: 18.5µl**   - 10µl RNA - 8.5µl of in-house fragmentation mix:   - 6.7µM DNA ready-made randomers (Integrated DNA Technologies)   - 1mM dNTPs (Invitrogen)   - 1X M-MLV reverse transcriptase buffer (Invitrogen)   Incubate at 85°C for 1 min |
| 1^st^ strand synthesis | **Final reaction volume: 30µl**   - 10µl 1^st^ strand synthesis mix:   - 11µl “1^st^ Strand Synthesis Buffer”   - 1µl KAPA Script - 20µl fragmented RNA | **Final reaction volume: 20.5µl**   - 2µl in-house 1^st^ strand synthesis mix:   - 1µl 0.1M DTT (Invitrogen)   - 1µl KAPA Script - 18.5µl fragmented RNA |
| 2^nd^ strand synthesis & A‑tailing | **Final reaction volume: 60µl**   - 30µl 2^nd^ strand & A-Tail mix:   - 31µl “2^nd^ Strand Marking Buffer”   - 2µl “2^nd^ Strand Synthesis & A-Tailing Enzyme Mix” - 30µl 1^st^ strand cDNA reaction | **Final reaction volume: 42.5µl**   - 22µl 2^nd^ strand & A-Tail mix:   - 20µl “2^nd^ Strand Marking Buffer”   - 2µl “2^nd^ Strand Synthesis & A-Tailing Enzyme Mix” - 20.5µl 1^st^ strand cDNA reaction |
| Adapter ligation | **Final reaction volume: 110µl**   - 45µl adapter ligation master mix:   - 40µl “Ligation Buffer”   - 10µl “DNA Ligase” - 5µl adapters (100nM) - 60µl 2^nd^ strand cDNA reaction | **Final reaction volume: 89µl**   - 40.5µl adapter ligation master mix:   - 30.5µl “Ligation Buffer”   - 10µl “DNA Ligase” - 5µl adapters (60nM) - 42.5µl 2^nd^ strand cDNA reaction |
| Post-ligation clean-up | 0.63X & 0.7X “KAPA Pure Beads” clean-ups | 0.78X & 0.7X “KAPA Pure Beads” clean-ups |
| Ligation-Mediated PCR | For 1-5 ng inputs, 11-15 PCR cycles | 18 PCR cycles |
