# Supplementary material for: Technical Validation of a Hepatitis C Virus Whole Genome Sequencing Assay for Detection of Genotype and Antiviral Resistance in the Clinical Pathway
Source: Front Microbiol. 2020 Oct 9;11:576572. doi: 10.3389/fmicb.2020.576572 (PMC7583327; doi:10.3389/fmicb.2020.576572)
Supplement: Supplementary Table S2 — Details of NS5A amplicon-based Sanger sequencing assay. [file Table_2.DOCX]

**Supplementary Table S2:** Details of NS5A amplicon-based Sanger sequencing assay

| **Gene target** | **Genotype** | **Reaction** | **Primers (sequence)** | **PCR cycling conditions** |
| --- | --- | --- | --- | --- |
| **NS5A** | **1a** | **RT-PCR** | 1a5A-3'7833 (5’-ACGGATAGCAAGTTAGCCTTCAC-3’); 1a5A-5’6095 (5’-GGCAGTGCAATGGATGAACCGG-3’) | RT: 43°C for 60min, followed by 70°C for 15min  PCR: Initial denaturation at 94°C for 2min; followed by 35 cycles of 94°C for 30sec, 54°C for 30sec, 68°C for 2min; and a final extension at 68°C for 5min |
|  |  | **Nested PCR** | 1a5A-3’7807 (5’-GACGCCGCTGCCTTAACCTCCT-3’); 1a5A-5'6217 (5’-CTCACTGTAACCCAGCTCCTGAGGCG-3’) | PCR: Initial denaturation at 94°C for 2min; followed by 35 cycles of 94°C for 30sec, 60°C for 30sec, 72°C for 2min; and a final extension at 72°C for 10min |
|  |  | **Sequencing** | 1a5A-3’6603 (5’-GCCTTATCTCCACGTATTCCTC-3’); 1a5A-5’6479 (5’-GTCAAAAACGGGACGATGAGGATC-3’); 1a5A-3’7050 (3’-CTGGTGATGTTGCCGCCCAT-5’) |  |
