# Supplementary material for: Technical Validation of a Hepatitis C Virus Whole Genome Sequencing Assay for Detection of Genotype and Antiviral Resistance in the Clinical Pathway
Source: Front Microbiol. 2020 Oct 9;11:576572. doi: 10.3389/fmicb.2020.576572 (PMC7583327; doi:10.3389/fmicb.2020.576572)
Supplement: Supplementary Table S3 — Variant frequency at WGS and Sanger discordant mixed base positions. [file Table_3.DOCX]

**Supplementary Table S3.** Variant frequency at WGS and Sanger discordant mixed base positions

| Gene region | Sample ID | Nucleotide bases | Amino acids | VF undetected base (%) | VF detected base (%) | Sanger:NGS |
| --- | --- | --- | --- | --- | --- | --- |
| NS5a | 17-1682 | 0 | 0 | - | - | - |
|  | 17-1772 | 6 | 2 | 12.4 ± 2.0 |  | 6 : 0 |
|  | 17-2157 | 3 | 0 | 4.9 ± 7.0 | 15.8 | 2 : 1 |
|  | 17-2173 | 11 | 2 | 13.2 ± 0.2 | 19.3 ± 4.1 | 2 : 9 |
|  | 17-2176 | 0 | 0 | - | - | - |
|  | 17-2291 | 0 | 0 | - | - | - |
|  | 17-2302 | 15 | 1 | 4.8 ± 4.9 | 28.1 ± 9.9 | 6 : 9 |
|  | 17-2303 | 2 | 1 | 13.0 | 19.4 | 1 : 1 |
|  | 17-2312 | 6 | 0 | 10.6 ± 2.1 | - | 6 : 0 |
|  | 17-2317 | 1 | 0 | - | 23.9 | 0 : 1 |
|  | 17-2318 | 0 | 0 | - | - | - |
|  | 17-2330 | 2 | 1 | 10.5 | 17.0 | 1 : 1 |
|  | 17-2334 | 1 | 0 | - | 19.1 | 0 : 1 |
|  | 17-2434 | 4 | 1 | 5.3 ± 6.9 | 66.4 | 3 : 1 |
|  | 17-2469 | 3 | 0 | - | 22.0 ± 8.3 | 0 : 3 |
| Total |  | 54 | 8 | 8.8% [2.5-13] | 19.8 [17-28.4] | 27:27 |
| NS5b | 12-0872 | 1 | 0 | 13.3 | - | 1 : 0 |
|  | 13-0626 | 2 | 1 | 2.6 | 32.5 | 1 : 1 |
|  | 13-0812 | 1 | 0 | 0 | - | 1 : 0 |
|  | 13-0493 | 0 | 0 | - | - | - |
|  | 13-0392 | 3 | 0 | 12.5 ± 2.0 | 16.5 | 2 : 1 |
|  | 13-0654 | 3 | 1 | 11.9 ± 0.9 | 17.9 | 2 : 1 |
|  | 14-0428 | 0 | 0 | - | - | - |
|  | 15-0209 | 0 | 0 | - | - | - |
|  | 16-0050 | 0 | 0 | - | - | - |
|  | 17-0712 | 1 | 1 | 13.0 | - | 1 : 0 |
|  | 17-0135 | 1 | 1 | 0 | 0 | 1 : 1* |
|  | 17-2304 | 0 | 0 | - | - | - |
| Total |  | 12 | 4 | 9.1% [0-13.5] | 32.5 [16.5-46.9] | 9:4 |

* This was a unique instance of complete discordance between two nucleotides at one site.
