# Supplementary material for: Technical Validation of a Hepatitis C Virus Whole Genome Sequencing Assay for Detection of Genotype and Antiviral Resistance in the Clinical Pathway
Source: Front Microbiol. 2020 Oct 9;11:576572. doi: 10.3389/fmicb.2020.576572 (PMC7583327; doi:10.3389/fmicb.2020.576572)
Supplement: Supplementary Table S4A — Comparison of expected and Splitpops outputs for pure subtypes synthetic data. [file Table_4.docx]

**Supplementary Table S4A.** Comparison of expected and Splitpops outputs for pure subtypes synthetic data

| Sample ID | Expected | | Results | | | |
| --- | --- | --- | --- | --- | --- | --- |
|  | Subtype | % reads | Subtype 1 | % reads | Other subtypes | % reads |
| #1 | 1a | 100 | 1a | 98.9 |  |  |
| #2 | 3a | 100 | 3a | 100 |  |  |
| #3 | 4a | 100 | 4a | 97 |  |  |
| #4 | 4a | 100 | 4a | 98.6 |  |  |
| #5 | 1a | 100 | 1a | 99.7 |  |  |
| #6 | 1a | 100 | 1a | 99.9 |  |  |
| #7 | 2a | 100 | 2a | 98.2 |  |  |
| #8 | 2a | 100 | 2a | 97.9 | Other gt2 | 1.9 |
| #9 | 1b | 100 | 1b | 99.9 |  |  |
| #10 | 1b | 100 | 1b | 99.2 |  |  |
| #11 | 2b | 100 | 2b | 99.7 |  |  |
| #12 | 2b | 100 | 2b | 99.6 |  |  |
| #13 | 2c | 100 | 2c | 96.7 | Other gt2 | 2.3 |
| #14 | 6 | 100 | 6 | 99.4 |  |  |
| #15 | 6 | 100 | 6 | 99.7 |  |  |
| #16 | 6 | 100 | 6 | 99.9 |  |  |
| #17 | 1a | 100 | 1a | 99.1 |  |  |
| #18 | 4a | 100 | 4a | 98.6 |  |  |
| #19 | 1a | 100 | 1a | 99.9 |  |  |
| #20 | 1a | 100 | 1a | 99.7 |  |  |
| #21 | 1a | 100 | 1a | 96.4 | 1c | 3.5 |
| #22 | 1b | 100 | 1b | 99.1 |  |  |
| #23 | 1b | 100 | 1b | 99.9 |  |  |
| #24 | 1b | 100 | 1b | 99.2 |  |  |
| #25 | 2a | 100 | 2a | 98.1 |  |  |
| #26 | 2a | 100 | 2a | 97.6 | Other gt2 | 2.2 |
| #27 | 2b | 100 | 2b | 99.7 |  |  |
| #28 | 2c | 100 | 2c | 96.5 | Other gt2 | 2.6 |
| #29 | 2c | 100 | 2c | 94.2 | Other gt2 | 4.9 |
| #30 | 3a | 100 | 3a | 100 |  |  |
| #31 | 3a | 100 | 3a | 99.8 |  |  |
| #32 | 3a | 100 | 3a | 99.4 |  |  |
| #33 | 4a | 100 | 4a | 97.3 | Other gt4 | 2.4 |
| #34 | 4a | 100 | 4a | 99.9 |  |  |
| #35 | 6 | 100 | 6 | 99.8 |  |  |
| #36 | 6 | 100 | 6 | 63.6 | HCV gt1-4  Unclassified | 9.2  26.0 |

**Supplementary Table S4B.** Comparison of expected and Splitpops outputs for mixed infection and recombinant virus synthetic data

| Category | Sample ID | Expected | | | | | Results | | | | |
| --- | --- | --- | --- | --- | --- | --- | --- | --- | --- | --- | --- |
|  |  | Subtype 1 | % reads | Subtype 2 | % reads | Ratio | Subtype 1 | % reads | Subtype 2 | % reads | Ratio |
| Mixed infections | #1 | 1b | 95.2 | 1a | 4.8 | 19.8 | 1b | 95.2 | 1a | 4.6 | 20.7 |
|  | #2 | 3a | 99.9 | 4a | 0.1 | 999.0 | 3a | 99.8 | - | - | - |
|  | #3 | 1a | 4.8 | 6 | 95.2 | 19.8 | 6 | 95.0 | 1a | 4.7 | 20.2 |
|  | #4 | 3a | 50.0 | 4a | 50.0 | 1.0 | 3a | 49.8 | 4a | 49.7 | 1.0 |
|  | #5 | 6 | 50.0 | 2b | 50.0 | 1.0 | 6 | 49.9 | 2b | 49.8 | 1.0 |
|  | #6 | 1a | 16.7 | 6 | 83.3 | 5.0 | 6 | 83.1 | 1a | 16.5 | 5.0 |
|  | #7 | 3a | 0.1 | 4a | 99.9 | 999.0 | 4a | 99.1 | - | - | - |
|  | #8 | 6 | 95.2 | 2b | 4.8 | 19.8 | 6 | 95.1 | 2b | 4.7 | 20.2 |
|  | #9 | 1b | 50.0 | 1a | 50.0 | 1.0 | 1b | 50.2 | 1a | 49.0 | 1.0 |
|  | #10 | 1a | 50.0 | 6 | 50.0 | 1.0 | 6 | 49.7 | 1a | 49.5 | 1.0 |
|  | #11 | 1b | 83.3 | 1a | 16.7 | 5.0 | 1b | 83.2 | 1a | 16.6 | 5.0 |
|  | #12 | 6 | 4.8 | 2b | 95.2 | 19.8 | 2b | 95.1 | 6 | 4.7 | 20.2 |
| Recombinant viruses | #1 | 1a | 64.2 | 2c | 35.8 | 1.8 | 1a | 71.0 | 2c | 27.3 | 2.6 |
|  | #2 | 1b | 66.9 | 2a | 33.1 | 2.0 | 1b | 68.3 | 2a | 30.4 | 2.2 |
|  | #3 | 1a | 64.7 | 2b | 35.3 | 1.8 | 1a | 70.0 | 2b | 29.7 | 2.4 |
|  | #4 | 1a | 76.9 | 1b | 23.1 | 3.3 | 1a | 62.9 | 1b | 35.4 | 1.8 |
|  | #5 | 1a | 76.2 | 1b | 23.8 | 3.2 | 1a | 62.1 | 1b | 36.0 | 1.7 |
|  | #6 | 1b | 86.3 | 1a | 13.7 | 6.3 | 1b | 74.4 | 1a | 24.9 | 3.0 |
|  | #7 | 1a | 95.0 | 1b | 5.0 | 19.0 | 1a | 97.1 | - | - | - |
|  | #8 | 1b | 64.3 | 2b | 35.7 | 1.8 | 1b | 69.0 | 2b | 30.2 | 2.3 |
|  | #9 | 6 | 64.5 | 2b | 35.6 | 1.8 | 6 | 67.9 | 2b | 30.0 | 2.3 |
|  | #10 | 6 | 86.5 | 6 | 13.5 | 6.4 | 6 | 80.6 | - | - | - |
|  | #11 | 4a | 74.1 | 1a | 25.9 | 2.9 | 4a | 76.9 | 1a | 21.2 | 3.6 |

**Supplementary Table S4C.** Comparison of expected and Splitpops outputs for pure subtype or mixed infections synthetic data combined with NHP background reads also containing human pegivirus.

| Category | Sample ID | Expected | | | | | Results | | | | |
| --- | --- | --- | --- | --- | --- | --- | --- | --- | --- | --- | --- |
|  |  | Subtype 1 | % reads | Subtype 2 | % reads | Ratio | Subtype 1 | % reads | Subtype 2 | % reads | Ratio |
| Single infections | #1 | 2a | 100 | - | - | - | 2a | 80.9 | - | - | - |
|  | #2 | 2a | 100 | - | - | - | 2a | 68.6 | - | - | - |
|  | #3 | 2a | 100 | - | - | - | 2a | 47.5 | - | - | - |
|  | #4 | 1a | 100 | - | - | - | 1a | 81.7 | - | - | - |
|  | #5 | 1a | 100 | - | - | - | 1a | 69.0 | - | - | - |
|  | #6 | 1a | 100 | - | - | - | 1a | 47.5 | - | - | - |
|  | #7 | 1b | 100 | - | - | - | 1b | 48.4 | - | - | - |
|  | #8 | 1b | 100 | - | - | - | 1b | 81.2 | - | - | - |
|  | #9 | 1b | 100 | - | - | - | 1b | 69.5 | - | - | - |
| Mixed infections | #10 | 1b | 95.2 | 1a | 4.8 | 19.8 | 1b | 65.6 | 1a | 3.5 | 18.7 |
|  | #11 | 1b | 95.2 | 1a | 4.8 | 19.8 | 1b | 46.2 | 1a | 2.4 | 19.3 |
|  | #12 | 1b | 95.2 | 1a | 4.8 | 19.8 | 1b | 78.5 | 1a | 4.0 | 19.6 |
